# Supplementary material for: Heritability of Immunity Traits and Resistance of Atlantic Salmon against the Sea Louse Caligus rogercresseyi
Source: Biology (Basel). 2023 Aug 2;12(8):1078. doi: 10.3390/biology12081078 (PMC10452322; doi:10.3390/biology12081078)
Supplement: Supplementary file 1 [file biology-12-01078-s001.zip › biology-2489351-supplementary.pdf]

Supplementary Material

Table S1. Primary antibodies used in indirect ELISA.

Marker	Source	Dilution	Reference
Nkef	Mouse	1:800	[1]
Tnfα	Mouse	1:500	[2]
Il-8	Mouse	1:500	[3]

A

Marker	Initial Position	Sequence	End Position
Nkef	169	HGEVCPAGWKPGSDTIKPD	187
Tnfα	161	TTPLSHIIWRYSDSIGVNA	179
Il-8	43	IGKLIKKVEM	52

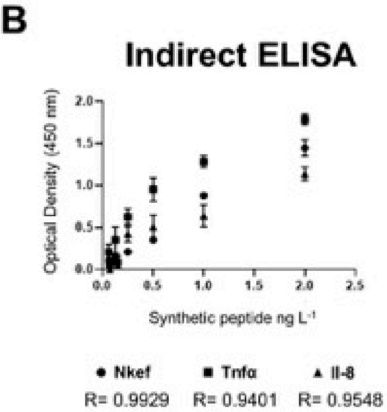

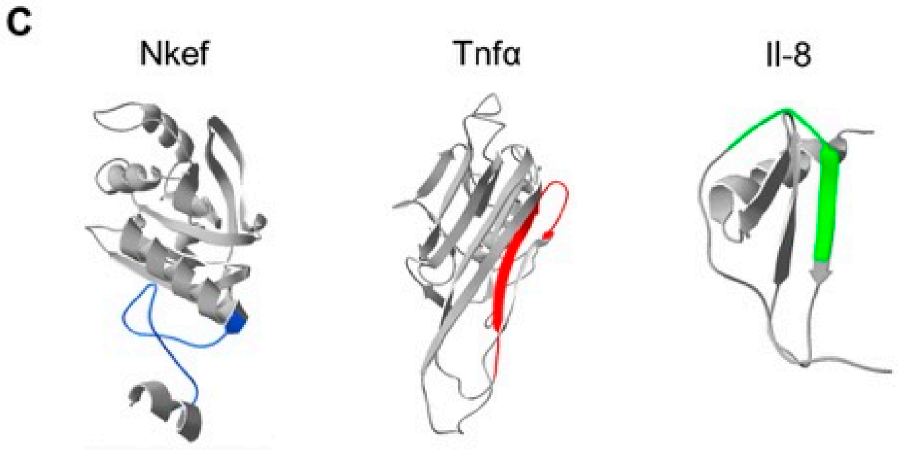

Figure S1. Production and validation of polyclonal antibodies against immunological markers of *Salmo salar*. (**A**) List of epitope peptides from Nkef, Tnf*α* and Il-8. (**B**) Calibration curve by indirect ELISA to establish the proportional relationship between the detection of the anti-epitope antibody produced (at 450 nm) and the concentration of the synthetic peptide (ng µL-1), R: Pearson's correlation coefficient. (**C**) Three-dimensional modeling of the epitope in the molecule by homology (Phyre2). On the left: Nkef (in blue: HGEVCPAGWKPGSDTIKPD), in the center: Tnf*α* (in red: TTPLSHIIWRYSDSIGVNA) and on the right.

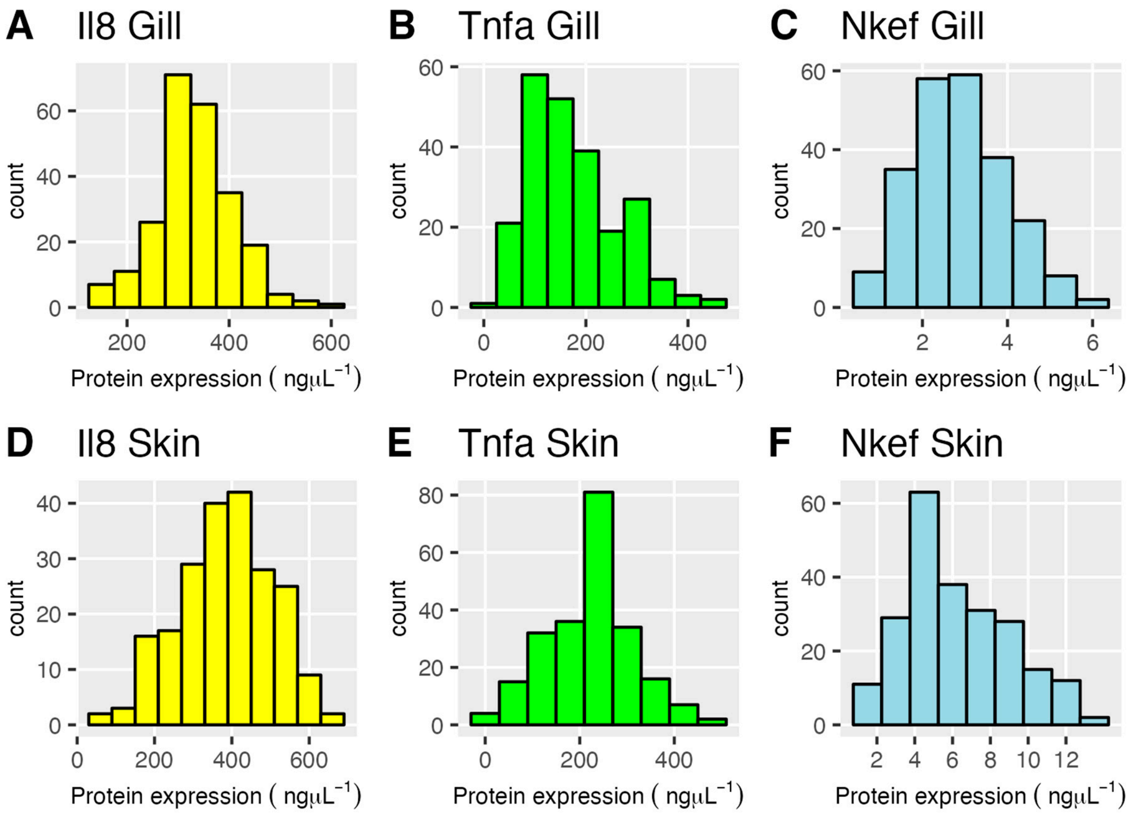

Figure S2. Phenotypic variation of immunity traits measured as Protein expression (ng μl-1) by indirect ELISA in salmon infected by sea lice (chalimus sessile lice) in Atlantic salmon.

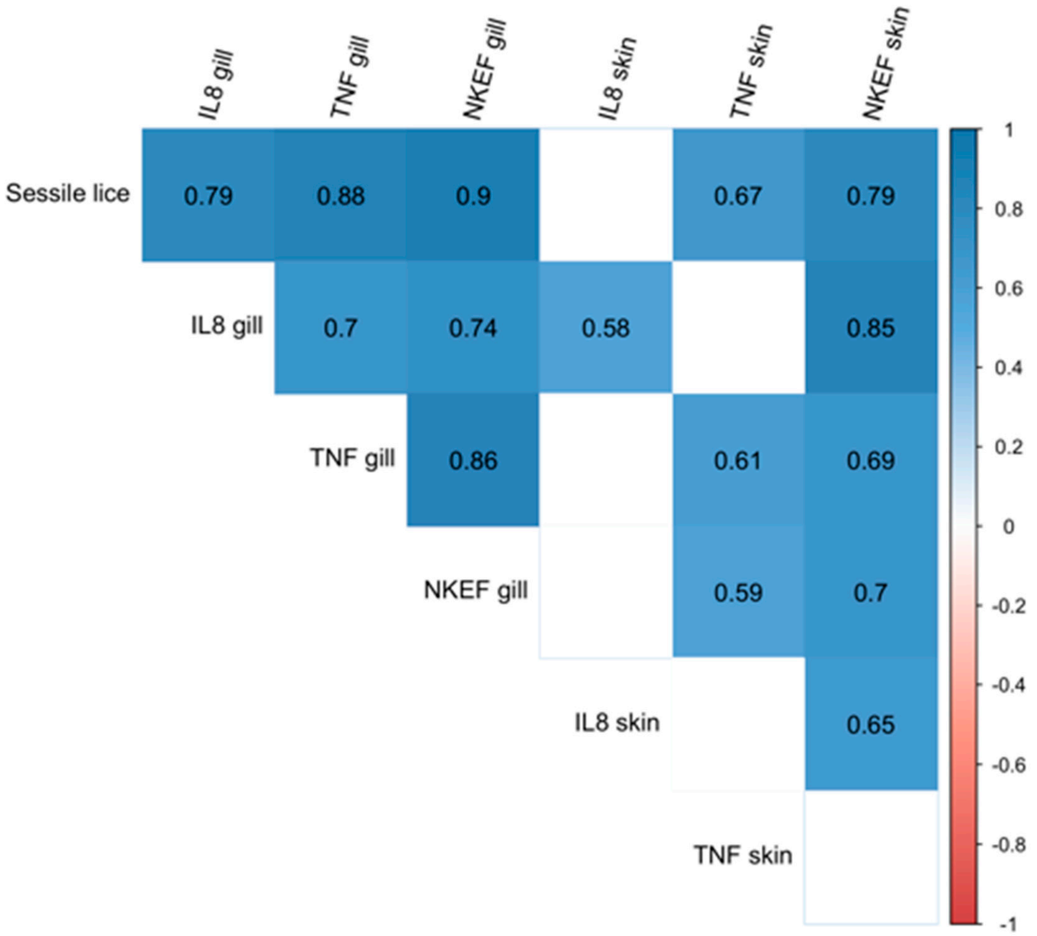

Figure S3. Significant ($p< 0.05$) familiar correlation of immunity traits measured as Protein expression (ng µl-1) by indirect ELISA in salmon infected by sea lice (chalimus sessile lice) in Atlantic salmon.

References

1. Bethke, J.; Rojas, V.; Berendsen, J.; Cardenas, C.; Guzman, F.; Gallardo, J.A.; Mercado, L. Development of a new antibody for detecting natural killer enhancing factor (NKEF)-like protein in infected salmonids. *Journal of fish diseases* **2012**, *35*, 379-388, doi:10.1111/j.1365-2761.2012.01354.x.
2. Rojas, V.; Morales-Lange, B.; Guzmán, F.; Gallardo, J.A.; Mercado, L. Immunological strategy for detecting the pro-inflammatory cytokine TNF-alpha in salmonids. *Electronic Journal of Biotechnology* **2012**, *15*, doi:10.2225/vol15-issue5-fulltext-19.
3. Santana, P.; Palacios, C.; Narváez, E.; Guzmán, F.; Gallardo, J.A.; Mercado, L. Anti-peptide antibodies: A tool for detecting IL-8 in salmonids. *Electronic Journal of Biotechnology* **2012**, *15*, doi:10.2225/vol15-issue5-fulltext-15.
